# Patients' Contributions to Hospitals

**Published:** 1888-06-16

**Authors:** W. Burdett-Coutts


### Patients' Contributions to Hospitals.

By Mr. W. Burdett-Coutts, M.P.

(*Continued from p.* 162.)

### Larger London Special Hospitals.

I now come to another class of London Hospitals—those intended for the treatment of special complaints. Amongst the fifteen of these special hospitals having over fifty beds, there are six which take paying patients; a very much larger proportion than in the case of the general hospitals. I cannot account for this. I am not aware that Nature in her choice of victims for special or general complaints makes any selection based upon the means of the patient. The most notable instance of the Pay System amongst the Special Hospitals is the London Fever Hospital, Liverpool Road, where, speaking generally, all are paying patients, whether in general wards or the private rooms. There are exceptions, twenty-nine in number last year, whose fees are remitted or reduced by the committee "to suit the circumstances of poorer patients, and those who lose their places when attacked with infectious fevers." While, therefore, there is a fixed charge for treatment, the committee do make some attempt to ascertain the means of the patient. Employés of subscribing firms, &c., are treated free in proportion to the sum subscribed annually, viz., one patient for every two guineas. The fee for the general wards is three guineas for the whole treatment, the average cost of which is £11 8*s*. The fee for a private room is three guineas per week. The total financial result of this system is so important that I give it in detail for three years.

| | Fees. | Cost. |
|---|---|---|
| 1885 | £2,180 | £8,543 |
| 1886 | 2,370 | 7,777 |
| 1887 | 3,873 | 9,932 |
| | £8,423 | £26,252 |

It will be seen that the patients pay about one-third of the cost of their treatment; the remaining two-thirds, with the exception of a very small endowment, being made up by charitable donations and subscriptions. This is more than the 25 per cent. which I have already suggested as a fair quota to be derived from patients' payments for the expenses of the hospital. The Secretary says he is "thoroughly satisfied" with the system: "It supplies efficiently a great public need by providing the means of isolation and treatment for persons of all ranks who would be unwilling to seek admission into the hospitals of the Metropolitan Asylums Board, which are intended for the reception of paupers, and which are controlled by the Poor Law authorities."

Although I am quite aware of the greater necessity for Hospital treatment in the case of infectious diseases, I cannot imagine why there should be no General Hospital in London worked upon the same admirable basis as the London Fever Hospital.

In the Chelsea Hospital for Women payments represent nearly a fourth of the expenses; and the report states of the patients, represented by 12,246 attendances, 7,593 out-patients "preferred to contribute towards the expenses of the hospital"; of 398 in-patients, 131 paid. The payment for out-patients (at sixpence per week without a "letter") amounted to £245; the payment for in-patients varies from ten shillings and sixpence to two guineas per week (the average payment being £4 1*s*. 8*d*. for thirty-six days), and this produced last year £535. Here there is a fixed payment, and no attempt is made to ascertain the means of patients.

I have mentioned these as specimens of the Pay System in the larger special hospital. I should be surprised to find that the class of patients treated at these particular hospitals differ very widely from those treated at the other special hospitals, and are not also at least included in the wide range of patients attending the large general hospitals.

### Smaller London Special Hospitals.

From the smaller special hospitals in London, those having less than fifty beds (it must be noticed that several of them have very large out-patient attendance), I have received fifteen answers; of these, six take no payment whatever, and nine take payment. The most noticeable results are obtained from the Pay System at the Hospital for Diseases of the Throat, Golden Square. Seventy-five per cent. of the patients pay, their payments amounting in 1887 to £2,021; as against an expenditure of £2,700. The payments are low, ten shillings and sixpence a week for in-patients, and one shilling, two shillings, or two shillings and sixpence a week for out-patients, which it is stated are fixed by "general inquiries from patients themselves." The London Throat Hospital, Great Portland Street, recently established, has adopted a judiciously-arranged Pay System, and during the first year of its existence has been very successful in maintaining it. An instance of this class of a more graduated Pay System is to be found in the Grosvenor Hospital for Women and Children, Vincent Square. Here all are paying patients and the payments are fixed by inquiries of those recommend, ing the cases, who are asked to write on the "letters" the sums which the patients are able to pay. The out-patients pay from twopence to one shilling per visit; in-patients pay ten shillings or five shillings per week in the general wards, and one-guinea a-week in the private wards. In-patients' payments amounted to £203; out-patients' to £205—together £408; against an expenditure of about £1,000. The Secretary, in answer to my inquiry, says: "The advantages: First, to the patient—this promotes a feeling of self-respect and prevents unnecessary attendance. Secondly, to the hospital—a considerable increase to the revenue, payments of 1887 amounting to nearly one-half of the receipts of the hospital." This financial result is very satisfactory, and the hospital appears to be well attended; but in contrast to this success I will now give another example.

### A Contrast.

In the Western Ophthalmic Hospital, the out-patients who are able to do so pay sixpence or one shilling per visit, the poor being taken free. In-patients, if able, pay a maximum sum of ten shillings and sixpence per week, sometimes reduced to five shillings. The means of the patient are stated to be ascertained as follows: "The patient registers name, address and occupation. If inquiry is desirable, it is done by two members of the committee, the 'visitors' for the month." The receipts for 1886 were: Out-patients, £144; in-patients, £34; total, £178; against an expenditure of £448

The Secretary appends the following interesting note: "Our committee are quite satisfied that their plan of requiring a small payment from those able to make it is right in every point of view, and that otherwise such persons (including many fairly 'well-to-do') would not hesitate to receive the benefit without any attempt to benefit the hospital. In its actual operation here, however, the result has been to largely diminish the attendance of patients, inasmuch as, of the five hospitals for the eye, this is the only one systematically requiring a small payment, and the medical staff are unanimous in objecting to the system."

This is only another proof of what I have already stated as to the necessity of the universality of the Pay System.

The other hospitals in this class from which I have received replies are treated in the table; but there is one, the Hospital for Epilepsy and Paralysis, Regent's Park, to which I would call especial notice, and from which I have received such a full and interesting reply that I have had it printed in a separate appendix (Appendix I.).

### Larger Provincial General Hospitals.

Of the larger provincial general hospitals, out of fifty-four which have sent replies, thirty-three do not take paying patients; and, of the remaining twenty-one, very few receive a contribution from this source equivalent to 10 per cent. of the expenses. The system, therefore, with some notable exceptions, to which I will allude, seems as little known in the larger hospitals of the country as in those of London, and as most of the former are situated in large towns, the same arguments for and against the Pay System in London would probably apply to them, with this exception, that if the patient's payment has to be fixed by a knowledge of his means, the provincial towns and cities, large as they may be, would, in comparison with London, afford greater facilities for the adoption of this method. In this class, the largest results are to be found in the Devonshire Hospital, at Buxton, which it is somewhat difficult to classify, but where the receipts for paying patients are £1,350, against an expenditure of £6,150. It is competent for any person to whom the patient is known to purchase a "casual recommendation," entitling the patient to remain for three weeks in the hospital for £2 12s. 6d., with the right to remain longer, if necessary, on payment of seventeen shillings and sixpence per week. The form of recommendation is intended to show that the patient is a fit object of charity. I am inclined to contrast this method favourably with the ordinary practice of governors' letters.

### Royal Albert Hospital, Devonport, and Others.

At the Royal Albert Hospital, Devonport, the Pay System is practically in force, as the out-patient department has been turned into a Provident Dispensary, and by this means has been rendered self-supporting, the receipts being £650. In-patients are taken, on payment, to the general and to the special wards, at a charge of £2 16*s.* per week for the special, and fourteen shillings for the ordinary wards. The receipts from both were about £100. The peculiarity of this case is that in the special ward the patients pay their own doctors, in addition to the payment to the hospital. The secretary, while remarking upon the success of the out-patient department, states that the special pay ward has not proved a success, but adds that they "have not a sufficient number of the class of persons likely to avail themselves of such accommodation resident there." At the Coventry Hospital £770 has been raised by systematic weekly collections in factories and workshops; but there is no statement of the privileges given in return for these, and the few patients that are taken on payment are domestic servants, etc. The Devon and Exeter Hospital do not take pay patients, but they have a regulation which carries out the principle of the system to the effect that—

"None are received as patients whose circumstances enable them to pay for medical attendance; nor the wives, children, nor apprentices of those who are able to provide for their medical relief; and if such shall have been admitted, they must pay to the hospital such sum or sums as the weekly board shall consider adequate."

At the Royal Southern, Liverpool, the Secretary says, as to the advantages of the system, "There is a difference of opinion on this point," which is somewhat surprising, when we find that they have only three pay beds, from which they received in 1887 £283, against an estimated expenditure of £194. A full special pay ward, carried on upon the same business like principles, might be expected to produce a profit of nearly £2,000 a-year to the hospital.

### Preston and County of Lancaster Infirmary.

In the Preston and County of Lancaster Infirmary pay patients are received for the same reason assigned by some other hospitals, "for the better performance of operations, the home surroundings not being suitable for such treatment, at a charge varying from five shillings to two guineas a-week, to be fixed by the board on a statement by the medical officer or recommending subscriber, giving the present income, number of family, etc., of the patient." But here, again, the real development of the pay system is to be found in the very large collections from mills and workshops, amounting to £1,800—nearly one third of the income of the hospital. In return for this, letters are given to the foremen, admitting one in and two out patients at a time, or six out-patients, for every three guineas.

Again, at the Clayton Hospital, Wakefield, the contribution from similar sources was over £900—one-fourth of the income of the hospital.

### Manchester Royal Infirmary.

I am indebted to the Secretary of the great Manchester Hospital, the Royal Infirmary, for an interesting statement of the method adopted at that institution to carry out the principles of the Pay System, and to check any improper use of the hospital. The payments amounted last year to £644 from in-patients, all treated in the general wards, and £124 from out-patients, the number of the former being 455, or 10 per cent. of the total number of in-patients, and the number of the latter being 544. The average cost of each in-patient treated is £2 12*s.* 3*d.*, and the average sum paid by each paying in-patient is £1 8*s.* 4*d.*, the latter, therefore, being made to pay slightly over one-half of the cost of his treatment. As to the method of inquiry, the Secretary states:—

"Inquiries are made in this infirmary into the circumstances of every in, out, and home patient, in the first instance by the Hospital Clerk, and, in a large number of cases, the result of these inquiries is verified by an officer of the Manchester and Salford Provident Society (the local Charity Organisation Society). The cases missed by the society are those coming from a distance. In-patients able to contribute towards their maintenance (however small the amount) are called upon to do so. Out and home patients residing in Manchester or Salford, whose earnings are found to be in excess of a fixed 'poverty' scale of wages, are refused further treatment after the first visit, and referred to the Provident Dispensary of the district in which they reside. The 'poverty' scale is eighteen shillings per week for a married couple, and one shilling and sixpence a week for each child; for an adult, twelve shillings per week. Out-patients residing out of Manchester, or where there is no Provident Dispensary, if found able to pay two shillings and sixpence a visit and upwards, are refused relief and referred to a private medical practitioner; if able to pay less than that sum, then a charge is made to them by the infirmary in accordance with their earnings."

He gives also the further information:—

"About fourteen years ago 'The Provident Dispensaries Association' was established in consequence of the enormous number of out-patients who sought relief from the Hospitals, and also to encourage habits of providence and self-help among the labouring classes. Members of these Provident Dispensaries are entitled (by agreement) to receive indoor treatment at this Infirmary, and also to superior advice in the case of out-patients—should their medical officer so recommend them. This organisation is unconnected with the Infirmary, but the latter works cordially with it."

The whole system gives satisfaction, and the advantages are stated to be—

"First, minimising imposition on the charity; secondly, the encouragement of provident habits amongst the labouring classes; thirdly, increased income in the form of contributions from patients who previously gave nothing at all."

At the Liverpool Royal Infirmary, with 258 beds, provision is to be made in the new building now being erected for the special accommodation of paying patients.

### Smaller Provincial General Hospitals.

Of the smaller general hospitals in the provinces, excluding cottage hospitals, out of twenty-one replies ten show no payment taken. Particulars of the remaining eleven will be found in the table, with some notes appended. The difficulties of ascertaining the means of the patients are seen to diminish according to the smaller populations.

The answer of the secretary of the Hospital at Stratford-on-Avon brings out one important feature of the Pay System, viz., that under that system a hospital would not be an almshouse, restricted to the absolutely poor, and excluding the large class of persons who are unable to pay the full cost of the best medical attendance iu their own homes.

"The advantages claimed are that people in a moderate position in life can have good medical attendance and skilled and properly trained nurses at a reasonable cost."

PROVINCIAL SPECIAL HOSPITALS.

Of the class of provincial special hospitals, thirty-seven replies give twelve that do not take paying patients. Amongst the remainder some very important financial results will be found on reference to the tables, the proportion of patients' payments to expenditure, averaged from fifteen of these hospitals, being 33 per cent.

At the Delancey Fever Hospital, Cheltenham, all patients pay, and yet "all classes enter it freely." The secretary sends an interesting statement of the graduated payments, the patients being allowed to choose which wards they will enter.

| | 1885. | 1886. | 1887. |
|---|---|---|---|
| | £ s. d. | £ s. d. | £ s. d. |
| Total receipts | 317 2 8 | 354 14 6 | 133 18 3 |
| No. of patients | 58, viz. :— | 34, viz. :— | 29, viz. :— |
| | At 10s. p. diem, 5. | At 10s. p. diem, 4. | At 1[illegible]s. p. diem, 4 |
| | " 2s. " 53. | " 5s. " 8. | " 5s. " 1 |
| | | " 2s. " 19. | " 2s. " 23 |
| | | Free from Gen. Hosp. 3. | Free from Gen. Hosp. 1 |

At the Bristol Hospital for Children and Women the payment is based on the income per head of the family, and is generally accepted on the patient's evidence. The receipts from out-patients for the past three years amount to £900, and the cost of the treatment of these paying patients to £1,300, leaving a net cost to the hospital of £400.

At the Rhyl Hospital for Children, patients pay £1,598, just half the expenditure of the institution. At the well-known National Hospital for Consumption, Ventnor, we find the combination of governors' letters and patients' payment, the latter source producing £2,400, against an expenditure of £8,700.

At the Birkenhead Fever Hospital, as at some other institutions, the Poor-Law Guardians pay twenty shillings per week for patients they send in—the system of relieving the sick poor adopted in Sweden and Norway. Ratepayers—and this is a novel criterion of means—pay an inclusive charge of fourteen shillings per week.

The Liverpool Eye and Ear Institution shows the existence of a central relief society, which makes inquiries when patients' statements are doubted.

MANCHESTER SOUTHERN HOSPITAL FOR WOMEN.

I have included the Manchester Southern Hospital for Women, with twenty-seven beds, in the list of hospitals that do not take paying patients, for the secretary states that there is no payment "beyond that out-patients pay for the bottles." But this happily appears to be very satisfactory, for "patients' pence" last year amounted to £217, and the whole cost of drugs and dispensary was only £199. These pence in this case covered more than half of the £394 cost of provisions of the hospital; it is 15 per cent. of their expenditure.

OPINION AT THE NOTTINGHAM SAMARITAN.

The secretary of the Nottingham Samaritan Hospital for Women suggests what would be a perfectly logical basis for all hospitals; but upon this point I would refer to the remarks I have already made as to hospitals being constituted almshouses pure and simple.

"Personally, I believe all hospitals should be *free* and confined solely to those who are unable to pay ordinary medical fees. Such hospitals as ours are much taken advantage of by people well able to pay, thus defrauding the medical profession, and in many cases preventing the admission of others whose circumstances should entitle them to gratuitous assistance."

SHEFFIELD SKIN HOSPITAL.

I cannot agree with the views of the Hospital Fund Committee in Sheffield, as referred to in the accompanying note from the secretary of the Sheffield Skin Hospital, unless they have positively assured themselves that in those hospitals which do participate in their fund no free patients are taken who can afford to contribute anything towards their own treatment.

"This hospital is managed by a committee. The Hospital Fund Committee in this town refused to allow this hospital to participate in the Hospital Fund *because* patients were allowed to contribute towards its support. At this hospital no patient is admitted who can afford to employ a medical man, while, at the others, *anyone* who can procure a recommendation may be admitted. Subscribers give recommendations indiscriminately, and well-to-do people apply and are admitted. Public dispensary constantly appeals to public for help, and threatens to shut up some beds, but will *not* allow patients to contribute. At all the other hospitals (except Ear and Throat) well-to-do people are constantly admitted on recommendation of careless and indolent subscribers."

SCOTCH HOSPITALS.

With respect to the Scotch Hospitals, from which I have received sixteen answers, details of which will be found in the tables, I have already referred to that form of the Pay System which consists in workmen's contributions. In the great industrial centre, Glasgow, this system produces larger results than are to be found anywhere else in Great Britain or Ireland. The Glasgow Royal Infirmary alone, with 542 beds, received last year £5,100 from this source. In return for these contributions, free letters are given in proportion to the amount subscribed. It seems to me that, if it is possible to develop this class of contribution to such an extent in a great city like Glasgow, it would not be impossible to do so in the East of London. At the Kilmarnock Infirmary, with 140 beds, an instance is found similar to that which obtains, but to a more limited extent, in some other hospitals, of a systematic arrangement between the parochial and the hospital authorities for the treatment of pauper patients. The parochial authorities give a donation of £100 per annum and the "Local Authority Board" £50 per annum; and when the number of patients sent by them exceeds respectively thirty-three and sixteen, the hospital charges for each patient beyond these numbers the usual rates—these are, to the general wards two shillings and sixpence per day. Patients wishing private treatment pay three shillings per day, and for this small extra charge may be treated in private rooms; but this can only be carried out to a limited extent. Other paying patients are admitted on subscribers' letters, and "the working classes subscribe very handsomely."

*(To be concluded.)*

